# From Herb to Hope: A Systematic Exploration of Medicinal Plants' Role in Cancer Therapy

**DOI:** 10.7150/jca.114837

**Published:** 2025-09-27

**Authors:** Sahar S. Alghamdi, Ruya Alshkrh, Afrah E. Mohammed, Shuroug A. Alowais

**Affiliations:** 1 King Saud Bin Abdulaziz University for Health Sciences (KSAU-HS), Riyadh, Saudi Arabia.; 2 King Abdullah International Medical Research Center (KAIMRC), Riyadh, Saudi Arabia.; 3 Ministry of National Guard Health Affairs (MNGHA), Riyadh, Saudi Arabia.; 4 Department of Biology, College of Science, Princess Nourah bint Abdulrahman University, Riyadh, Saudi Arabia.; 5 Microbiology and Immunology Unit, Natural and Health Sciences Research Center, Princess Nourah bint Abdulrahman University, Riyadh, Saudi Arabia.

**Keywords:** *Saussurea costus*, *Lepidium sativum*, *Rhus tripartite*, *Pyrus communis*, *Chenopodium murale*, *Erucaria hispanica*, *Trigonella hamosa*, *Argemone ochroleuca*, *Galium odoratum*, anticancer, *in vitro*, *in vivo*

## Abstract

Medicinal plants play a critical role in drug development, serving as a valuable source of bioactive compounds. Cancer, characterized by uncontrolled cell proliferation, presents significant challenges in treatment due to its multifaceted nature. This study aims to evaluate the anticancer potentials of selected medicinal plants specifically focusing on *in vitro* and *in vivo* studies that evaluate therapeutic implications for cancer treatment. A systematic review was conducted to assess both in *vitro* and *in vivo* studies involving selected medicinal plants:* Saussurea costus*, *Lepidium sativum*, *Rhus tripartite*, *Pyrus communis*, *Chenopodium murale*, *Erucaria hispanica*, *Trigonella hamosa*, *Argemone ochroleuca*, and *Galium odoratum.* The review involved analyzing cancer cell lines, plant parts used, extraction methods, and mechanisms of action reported in the literature. A total of sixty-nine articles were identified that investigated the anticancer properties of the selected plants. Notably, *S. costus*, *L. sativum*, and *R. tripartite* exhibited significant anticancer potential. In contrast, *P. communis*, *C. murale*, *E. hispanica*, *T. hamosa*, *A. ochroleuca*, and *G.odoratum* had limited studies available. The predominant mechanism of action identified for the anticancer activity was the induction of apoptosis. The findings indicate that these medicinal herbs possess promising therapeutic potential as anti-cancer agents. However, further research is warranted for *P. communis*, *C. murale*, *E. hispanica*, *T. hamosa*, *A. ochroleuca*, and *G. odoratum* to enhance understanding of their anticancer activities and explore their full therapeutic capabilities.

## 1. Introduction

Medicinal plants have played an important role in the discovery of innovative treatments for a variety of diseases. Compounds derived from many herbal plants have demonstrated important therapeutic effects on human pathologies 1. As a result, medicinal plants are increasingly recognized as a potential source for identifying candidate drugs, particularly in the search for cancer therapies and in efforts to minimize cancer cell resistance to treatment.

Cancer is one of the diseases that has been an obstacle in the scientific and medical fields due to its complex biological nature. The use of plants as potential cancer therapies has been a subject of interest throughout history 2. Globally, approximately 20 million new cancer cases and 9 million cancer-related deaths were reported in 2022 3. The treatment of these diseases is becoming more challenging due to resistance to current treatments. In addition to resistance, the side effects of the cancer therapies impact the patient's quality of life. Thus, it is a global concern, as it can hinder treatment and has an impact on the prognosis of the disease, prompting researchers to pursue novel pharmacological solutions.

In recent years, there has been an ongoing quest for effective drugs as a preventive therapies for cancer, and to overcome the resistance issue 4. In this context, medicinal plants are being studied as possible anticancer treatments. Recent findings on medicinal plants (*Saussurea costus, Lepidium sativum, Rhus tripartite, Pyrus communis, Chenopodium murale, Erucaria hispanica, Trigonella hamosa, Argemone ochroleuca,* and* Galium odoratum)* have increased the scientific interest in their bioactive compounds, particularly anticancer properties 5-13. Traditionally, these plants have been used for a variety of purposes, including antimicrobial, antioxidant, and anticancer applications. They contain a variety of secondary metabolites, such as polyphenols, flavonoids, steroids, which enhance their ability to induce the cancer cell death via different mechanisms.

The main aim of this systematic review is to assemble all current studies that explore and analyze the anticancer activity of the extracted parts of the selected medicinal plants in both *in vitro* and* in vivo* models.

## 2. Methods

### 2.1. Research questions

**Figure 1:**
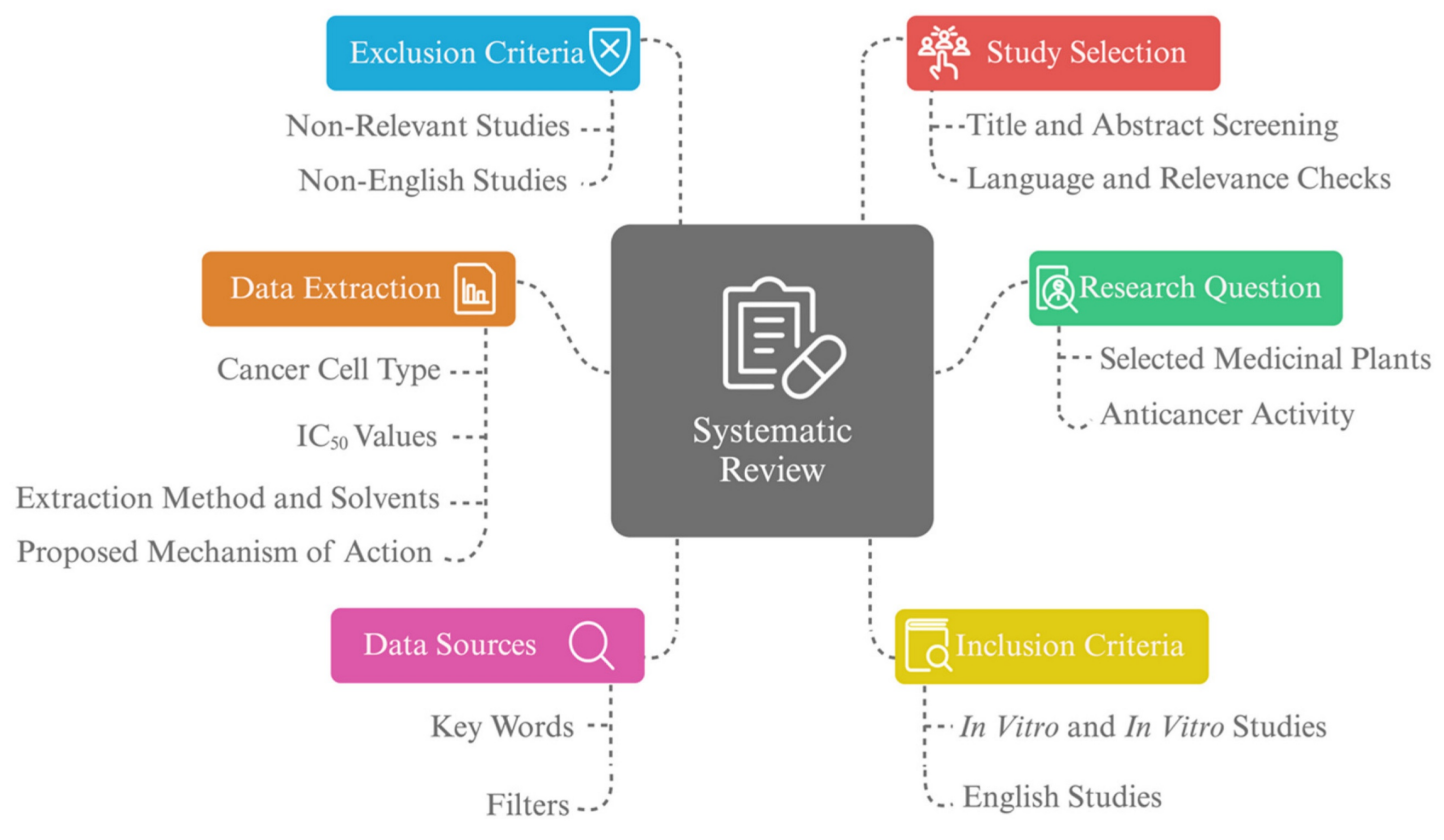
** The systematic review approach and scope.** A systematic review focused on the anticancer activity of selected medicinal plants, including original studies such as *in vitro* and *in vivo*. Data selection was based on the study's title, language, and relevance objectives, with data sources determined by keywords and filters. Data extracted includes cancer cell type, extraction methods, IC_50_ values, and proposed mechanism of action.

This systematic review focuses on the analysis of the anticancer activity for the extracts or isolated compounds of selected plants (*Saussurea costus, Lepidium sativum, Rhus tripartite, Pyrus communis, Chenopodium murale, Erucaria hispanica, Trigonella hamosa, Argemone ochroleuca,* and* Galium odoratum)* and their effects on *in vitro* and *in vivo* models, following the Preferred Reporting Items for Systematic Reviews and Meta-Analyses (PRISMA) guidelines. We included studies published within the past 24 years (2000-2024) to ensure adequate representation of historical and recent research findings regarding the selected medicinal plants (Figure 1).

### 2.2. Data sources

Google Scholar was utilized as a database to identify the studies that investigate the anticancer properties of the selected medicinal plants. As a search strategy, to conduct a comprehensive search, we utilized certain keywords and specific phrases, it involves “*Saussurea costus*" AND ("anticancer" OR "cytotoxic") AND ("*in vitro*" OR "*in vivo*"), "* Lepidium sativum*" AND ("anticancer" OR "cytotoxic") AND ("*in vitro*" OR "*in vivo*"), and so forth for each plant. The search is limited by the filter to include the articles that are published between 01/01/2000 to 01/08/2024 in English.

### 2.3. Inclusion criteria

Selected articles were required to investigate the potential anticancer effects for the chosen medicinal plants using *in vitro* or *in vivo* models, with plant extracts or isolated compounds tested against cancer cell lines or tumor-induced animal models.

### 2.4. Exclusion criteria

Articles were excluded if they did not evaluate anticancer activity or did not involve cancer cell lines or tumor models. Additionally, non-English articles, clinical trials, and non-original studies (e.g., systematic reviews or meta-analysis) were excluded.

### 2.5. Study selection

**Figure 2:**
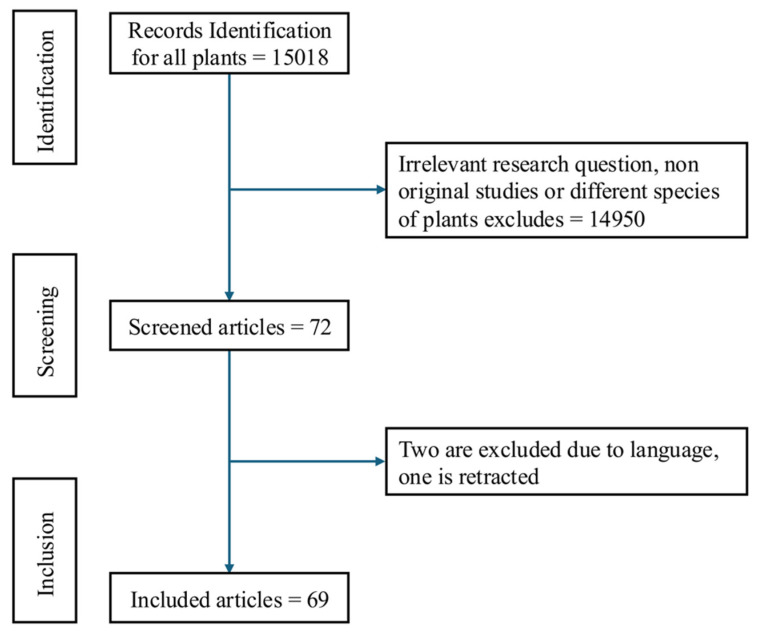
** Flow chart illustrating the study selection process:** A total of 15,018 records were identified, of which 14,950 were excluded due to irrelevant research questions or different plant species. After screening 72 records, 2 were excluded due to language barriers and one was retracted resulting in 69 studies included in the final analysis.

The screening of the preselected articles, using the described search strategy, was conducted by a single author and initially based on title and abstract review (Figure 2). Duplicates, studies irrelevant to the research question, and those investigating different plant species were excluded. Subsequently, the studies that are written with different languages other than English were eliminated. The final selection of the eligible studies was carried out by a single author.

### 2.6. Data extraction

Data extraction was carried out by a single author for all included articles. The extracted information encompassed several key aspects: the type of cancer cells treated with the plant extracts, the specific plant name, the part of the plant utilized in the study, the extraction methods and solvents employed by researchers, and the results, including values for IC_50_. Additionally, any proposed mechanisms of action suggested by the studies were recorded.

## 3. Results

To assess their anticancer activity and mechanism of action, a comprehensive review of *in vitro* and *in vivo* studies on nine plants— *Saussurea costus*, *Lepidium sativum*, *Rhus tripartite*, *Pyrus communis*, *Chenopodium murale*, *Erucaria hispanica*, *Trigonella hamosa*, *Argemone ochroleuca*, and *Galium odoratum* —was conducted. The selected medicinal plants have been investigated for their cytotoxic potential against a variety of cancers, including acute myeloid leukemia, breast cancer, lung cancer, colon cancer, tongue squamous cell carcinoma, colorectal cancer, liver cancer, prostate cancer, melanoma, cervical cancer, neuroblastoma, adenocarcinoma, ovarian cancer, esophageal cancer, gastric cancer, and skin cancer. Following a thorough examination for sixty-one *in vitro* and *in vivo* studies, the findings revealed that the *S. costus*, *L. sativum*, *R. tripartite*, *C. murale*, *P. communis*, *E. hispanica*, *T. hamosa* were investigated for their cytotoxic activity. No studies were found evaluating the anticancer properties of *A. ochroleuca* and *G. odoratum*s' anticancer properties (Table 1).

The proposed mechanism of action is mostly related to generation of reactive oxygen species (ROS) and induction of apoptosis.

### 3.1. *Saussurea costus*

*S. costus* was the most extensively studied plant across diverse types of cancer, including breast cancer (MCF-7, MDA-MB-231, SK-BR-3, MDA-MB-453), colon cancer (HTC116, Caco2, LS174T, HT-15, HT-29), liver cancer (HepG-2, HuH-7, PLC/PRF/5, SMMC-7721), lung cancer (A549, SK-MES-1), gastric cancer (AGS), central nervous system (CNS) cancer (XF498, IMR-32, SH-SY5Y, Rat B103), prostate cancer (PC-3, LNCaP, DU145), leukemia (HL-60, Jurkat E6-1, THP-1), cervical cancer (HeLa), esophageal cancer (Eca109, KYSE150), ovarian cancer (SK-OV-3, OVCAR3), soft tissue sarcoma (SW-872, SW-982, TE-671) 14-33.

*In vitro* studies utilized different parts of *S. costus;* however; root was the most utilized part for the most cancer types, while a few studies tested leaves and fruits of *S. costus* for breast (MCF-7), colon (CaCo-2), liver cancer (HepG2) 22,25,29,34-38. Other studies investigated isolated compounds, namely costunolide and dehydrocostuslactone 14-16,18,20,21,32,39-41.

Breast cancer was the most studied cancer type, with MCF-7 as the predominant cell line, in addition to MDA-MB-231, SK-BR-3, and MDA-MB-453, demonstrating the versatility of extracts and solvents. In a study conducted by Peng et al. (2013), the roots were extracted using liquid-liquid extraction with methanol and ethyl acetate and tested on MCF-7 cell lines, revealing IC_50_ values ranging from 1.7 to 6.1 μg/mL^28^. Similar findings were reported by Bhushan et al. (2023), where liquid-liquid extraction was applied to root extracts and tested on the MDA-MB-231 cell line using additional solvents such as hexane, chloroform, ethanol, and butanol^37^. A similar trend was observed, where hexane and chloroform showed IC_50_ values of 5.3-12.18 μg/mL, whereas ethanol and butanol exhibited higher IC_50_ values, ranging from 20 to >100 μg/mL. Comparable results were observed for other plant parts, including leaves and fruits, as summarized in Table 2.

Additionally, other studies have explored alternative extraction techniques, such as nanoparticle synthesis and supercritical carbon dioxide extraction, to evaluate the antitumor potential of *S. costus* extracts 14,42,43. One study optimized the extraction of *S. costus* oil using supercritical fluid extraction at different pressures, achieving significant inhibition with an IC_50_ value of 0.46 μg/mL on MCF-7 cells 42. In contrast, magnesium oxide nanoparticles synthesized from *S. costus* methanol extracts exhibited relatively lower antiproliferative effects on MCF-7 cells, with IC_50_ values of 80 μg/mL and 26.7 μg/mL for magnesium oxide and palladium nanoparticles, respectively 14,42.

Choi, (2009) tested dehydrocostuslactone on MDA-MB-231, SK-BR-3, and MDA-MB-453 cell lines, reporting IC_50_ values ranging from 25.6 to 43.2 μM/mL, while costunolide tested on MCF-7 cells showed an IC_50_ value of 30.16 μM/mL 39.

A similar pattern of inhibition, depending on plant parts, solvents, and extraction methods, was observed in other cancer types, including colon, liver, gastric, esophageal, and pancreatic cancers 14,28,28,30,37,43,43-45. Although hexane and chloroform extracts generally showed potent inhibition, one study found no significant cytotoxicity with hexane root extracts against colon (HCT-116, HCT-29) and prostate cancer (PC-3, LNCaP, DU145), as the hexane extracts did not affect cell viability 21,38.

However, neuroblastoma cell lines (XF498, SH-AY5Y, and B103) showed susceptibility to both methanol and ethanol extracts of *S. costus*, with IC_50_ values ranging from 15-20 μg/mL and 0.43-1.70 μM/mL when exposed to ethanol and methanol extracts, respectively 29,46.

Other cancer types that demonstrated sensitivity to *S. costus* extracts include ovarian, colorectal, skin, and soft tissue cancers 15,22,24.

**Figure 3:**
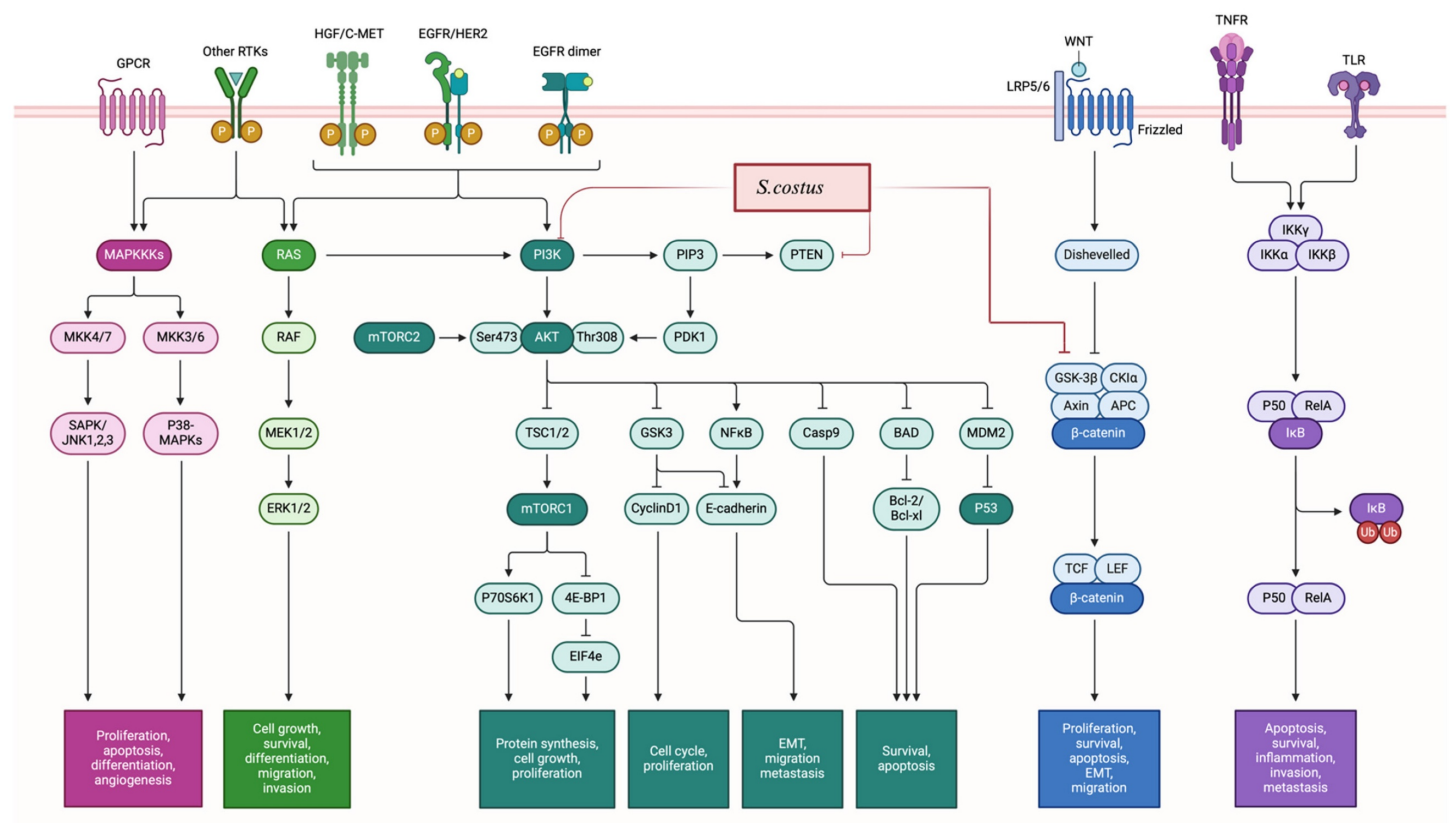
** Proliferation inhibition:** The mechanisms of action of *S. costus* involved the modulation of the PI3K/AKT/mTOR pathway, as well as the regulation of the MAPK/ERK pathway. Additionally, *S. costus* impacts the WNT/β-Catenin pathway by reducing β-catenin levels and depicts the inhibition of the NF-κB pathway. Furthermore, the regulation of cell cycle proteins, such as CDKs and cyclins leading to induction of cell cycle arrest.

The cytotoxic potential of* S. costus* has been extensively investigated, and it has been observed that its anticancer effects are mediated through multiple mechanisms (Figure 3), including the induction of apoptosis, cell cycle arrest, modulation of the androgen receptor and autophagy processes, decreased proliferation, and alterations in cellular signaling cascades by modulating phosphorylation processes. In particular, autophagy appears to be promoted through upregulation of LC3I and LC3II levels (high LC3II/LC3I ratio) and beclin1, while mTOR phosphorylation is inhibited 32,33,47. The interplay between autophagy and apoptosis is notable; inhibition of one can stimulate the other, suggesting a dual mechanism of action.

Ten *in vivo* studies have evaluated the anticancer activity of *S. costus.* The investigated type of cancer included breast cancer (MCF-7, MDA-MB-231), liver cancer (SMMC-7721), lung cancer (LC-540), leukemia, laryngeal carcinoma, esophageal cancer, gastric cancer (MKN-28) 47-51 (Table 3). Most studies utilized either the roots or isolated compounds (costunolide and dehydrocostus lactone), with ethanol and hexane as organic solvents for the extraction.

One study found that hexane root extract of* S.costus* inhibited hepatocellular carcinoma (HCC) with an inhibition rate of 55.71% 52. Likewise, ethanol extracts showed anti-leukemic effects by reducing white blood cell counts to normal levels 50.

The isolated compounds also exhibit anticancer activity, the lowest dose (costunolide at 10 mg/kg/day) was tested on immunodeficient female NCr nude homozygous mice with breast and colon cancer, showing reduction of the tumor volume. The highest dose (dehydrocostus lactone at 40 mg/kg/day for 28 days) was tested also for esophageal cancer in the Eca109-b mouse model, and the results showed inhibition of the tumor growth 27. Several studies attempt to investigate the mechanisms, the *in vivo* anticancer activity of *S. costus* is related mainly to induction of apoptosis by activating pro-apoptotic proteins such as p53 and Bax, while inhibiting anti-apoptotic proteins like Bcl-2, leading to programmed cell death, cell cycle arrest, modulating certain signaling pathways, such as EGFR, which reduces the proliferation and invasion of cancer cells, and suppress PI3K/Akt and MEK/P38 pathways, reduction the inflammatory process by suppression the level of TNF-α and NF-κB, and induction of reactive oxygen species.

### 3.2. *Lepidium sativum*

Fourteen studies have investigated the anticancer activity of *L. sativum* on various cancer types, such as liver (HuH-7 and HEPG-2), breast cancer (MCF-7), colon cancer (DLD-1, HCT-116, HT-15, HT-29, SW480, HTB-38, and Caco2), cervical cancer (HeLA 2), lung cancer (A-549 cell line), prostate cancer cells (P-C3), endometrium cancer (ECC-1), tongue squamous carcinoma (CAL-27), melanoma cancer (A-375 cell line), neuroblastoma (IMR-32), ovarian cancer (OV17R), leukemia (Jurkat E6-1)^53^-^66^. The studies utilized various plant parts, including leaves, roots and seeds, with th most employed seeds being the most commonly used part.

Liver cancer was the most commonly studied cancer type. Studies reported similar IC_50_ values across different cell lines, plant parts, and extraction solvents 58,64, particularly when polar solvents were used. This pattern was also observed in other cancer types, including breast, colon, cervical, lung, prostate, endometrial, tongue squamous cell carcinoma, and leukemia 54,55,57,60,66. However, the results showed variability in cytotoxic activity.

In a study by Abd-elmegeed et al. (2023), phenolic compounds, such as rutin, benzoic acid, cinnamic acid, and vanillin were isolated and evaluated, revealing IC_50_ values ranging from 28.8 μg/mL to 64.32 μg/mL 53. Furthermore, Ibrahim et al. (2023) demonstrated that *L. sativum* ethanol extracts that are treated with glucosinolates showed inhibition of cancer cells, prostate cancer (PC-3), colon cancer (coca2), lung cancer (A-549), and liver cancer (HepG2) with IC_50_ ranges 38.5-92.6 mg/mL, without effect normal cell lines 59.

Nanoparticles synthesis using *L. sativum* extracts has also been explored. Meer et al. (2022), and Efati et al. (2023) reported enhanced cytotoxic potential of *L. ativum* extracts against colorectal adenocarcinoma (SW480) and colon adenocarcinoma (HT-29 and Caco-2) at different degrees of temperature, the lowest IC_50_ (13.14 µg/mL) was detected to SW480 with ZnO at 350 °C 57,63. In contrast, Amina et al., (2021) study of Ag-MgO nanoparticles found no enhancement of *L. sativum* extracts 55.

**Figure 4:**
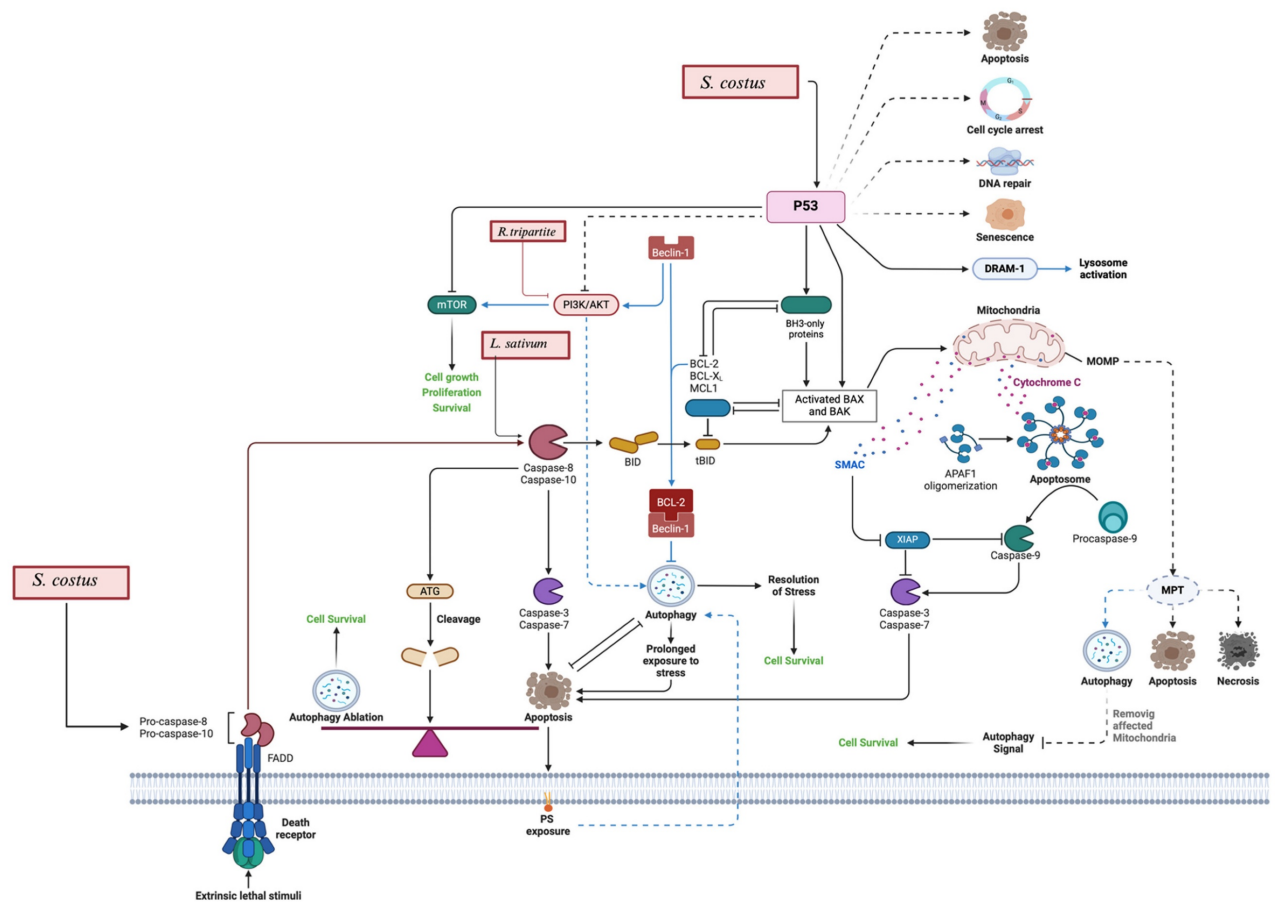
** Apoptosis and autophagy:** *S. costus* and *L. sativum* activate p53, which leads to DNA damage response, cell cycle arrest, and apoptosis by upregulating pro-apoptotic proteins like Bax and Bak while down regulating anti-apoptotic proteins such as Bcl-2 and Mcl-1. Additionally, *S. costus* influences autophagy pathways, including Beclin-1 and the interaction of death receptors, leading to autophagic cell death. It includes also the apoptosis by ROS generation, which leads to mitochondrial dysfunction and apoptosis. *R. tripartite* is also involved in the modulation of the PI3K/AKT/mTOR pathway.

Several studies have investigated the mechanism of action underlying the anticancer effects of *L. sativum*. These studies demonstrated that the anticancer activity is related mainly to induction of apoptosis and cell cycle arrest. *L. sativum* extracts showed upregulation of pro-apoptotic proteins such as BAX, p53, and caspases 3/7, alongside the downregulation of anti-apoptotic proteins like Bcl-2 (Figure 4) 57. Additionally, upregulation of SMAD2 and SMAD3 expression has been observed in the live cancer cell upon exposure to *L. sativum* extracts. Moreover, *L. sativum* extracts showed induction of cell cycle arrest at the S phase, thereby inhibiting their proliferation (Table 4) 59.

### 3.3. *Rhus tripartite*

The cytotoxic potential of *R. tripartite* leaves and roots was examined in four studies, using diverse cancer cell lines, including acute myeloid leukemia (THP-1), myelogenous leukemia (K-562), colon cancer (DLD-1, Caco-2), breast cancer (MCF-7), lung cancer (A-549) (Table 6) 67-71. The extraction process used butanol, methanol, ethanol, and ethyl acetate as organic solvents. The lowest IC_50_ value (39.83 μg/mL) was reported for methanol extracts against colon adenocarcinoma DLD-1 cell line 68.

In contrast, aqueous extracts showed lower cytotoxic activity with IC_50_ values 195.37 μg/mL and 200 μg/mL against A-549 and DLD-1) cell lines, respectively. Similarly, in Tlili et al. (2019) reported IC_50_ values less than 50 μg/mL for methanol extracts against CaCo-2 and K-562 cell lines 71.

The mechanism of action for *R. tripartite* extractions is thoroughly explained by Tlili et al., 2021, which involves the inhibition of the phosphatidylinositol 3-kinase (PI3K)/protein kinase B (AKT mammalian target of rapamycin (mTOR) pathway that eventually results in the induction of apoptosis and the suppression of tumor growth, as shown previously in Figure 4 70.

### 3.4. *Pyrus communis*

Cytotoxic potential of *P. communis* has been examined in two studies on various cancer types, including lung cancer (A549, WI-38), prostate cancer (LNCaP), urinary bladder cancer (HCV29T), kidney cancer (A-498), mouse myelogenous leukemia carcinoma (M-NFS-60), ovary cancer (CHO-K1) 72,73.

Both studies utilized the fruits of *P. communis*. The study that utilized hydroinstillation extraction method showed higher cytotoxicity, with IC_50_ values ranging values: from 30.9 to 105 μg/mL. In contrast, the study employing the UPLC-PDA-MS extraction method showed lower cytotoxicity, with IC_50_ values ranging from: 0.5 - to 3.2 mg/mL (Table 7) 72,73.

### 3.5. *Chenopodium murale*

Only one *in vitro* study has evaluated *C. murale's* anticancer activity in breast cancer (MCF-7) and liver cancer (HCAM), with leaves extracted using an ethanol solvent. The investigation found that the extraction had weak cytotoxic activity, with IC_50_ of 1504 µg/mL for breast cancer and 1267 µg/mL for liver cancer cells (Table 8) 74.

### 3.6. *Erucaria hispanica*

*E. hispanica* was investigated in one study involving four different cancer cell lines, breast (MCF7), liver (HEPG2), cervix (HELA) and colon (HCT116) cancers, utilizing methanol extracts of aerial parts of *E. hispanica* 10.The results showed IC_50_values 18 μg/mL, 20.8 μg/mL, 14.7 μg/mL and 21.4 μg/mL respectively (Table 9).

### 3.7. *Trigonella hamosa*

A single study has investigated to explore the anticancer activity of methanol extracts from *T. hamosa* aerial parts. The reported IC_50_ values were 6.71 μg/mL for breast cancer (MDA-MB-231), 4.93 μg/mL for lung cancer (A549), and 13.74 μg/mL for colon cancer (HTC-166) (Table 10) 75.

## 4. Discussion

Medicinal plants have long served as a valuable source for the discovery of novel therapeutic agents, particularly in the treatment of diseases such as cancer 76. Cancer remains a complex and life-threatening condition, contributing to rising mortality rates globally. Additionally, the development of resistance to existing treatments further complicates cancer management and presents significant challenges 77. Consequently, there is a pressing need to identify new therapeutic agents that can enhance current treatment strategies and address resistance issues. Several medicinal plants have shown promising therapeutic properties, including anticancer activity.

This systematic review critically assessed the available evidence on the anticancer potential of selected medicinal plants. A total of sixty-nine studies were identified that investigated the anticancer effects of for *S. costus, L. sativum, Rhus tripartite, C. murale, P. communis, E. hispanica, T. hamosa.* In contrast, *A. ochroleuca, and G. odoratum* have not yet been evaluated for their anticancer potential.

Among these, *S. costus* was the most extensively studied. It has been tested against various cancer types, most notably breast (MCF-7, MDA-MB-231, SK-BR-3), liver (HepG2), and colon cancers (HCT-116, HT-29). The cytotoxic activity of *S. costus* varied depending on the extract type and cell line. For instance, non-polar organic solvents like hexane and chloroform demonstrated high potency, with IC_50_ 0.4 µg/mL - 2.1 µg/mL 30. However, this potency did not consistently extend to all cancers—prostate cancer cell lines (PC-3, LNCaP, DU145), for example, showed resistance to hexane extracts 21,38. In comparison, extracts prepared with polar solvents (methanol, ethanol, butanol) generally showed lower potency (IC_50_ values:10 µg/mL to > 100 µg/mL), suggesting solvent polarity significantly influences the bioactivity of phytoconstituents present in *S. costus* 37,45,78. In contrast, other cancer types showed to be sensitive, such as ovarian, colorectal, skin, and soft tissue, regardless of polarity of the solvents or extraction method.

Skin cancer (SK-MEL-2) cells showed exceptional sensitivity to costunolide and dehydrocostus lacton, two well-characterized sesquiterpene lactones isolated from *S. costus*, with IC_50_ ranging from 4.7 µM to 60 µM. These compounds demonstrated consistent tumor-suppressive activity across various studies. Advanced extraction techniques like supercritical CO₂ extraction yielded highly potent results, with IC₅₀ values of 0.44-0.74 μg/mL against HCT-116, MCF-7, and HepG2 cells 42. Nanoparticle formulations also enhanced cytotoxicity: methanolic *S. costus*-derived palladium nanoparticles showed IC₅₀ values of 7.8-26.7 μg/mL, whereas magnesium oxide nanoparticles were moderately effective (IC₅₀ = 80 μg/mL) 14,43. These findings highlight that supercritical extraction and specific nanoparticle formulations can optimize *S. costus's* therapeutic potential.

*In vivo* studies support the anticancer potential of *S. costus*: hexane extracts demonstrated a 55.71% inhibition rate on hepatocellular carcinoma (HCC) 52. While ethanol extracts effectively normalized white blood cell counts in leukemia 50. Costunolide (10 mg/kg/day) reduced breast and colon tumor volumes in mice, while dehydrocostuslactone (40 mg/kg/day) inhibited esophageal tumor growth 78. These findings reinforce the therapeutic relevance of *S. costus*.

Mechanistically*, S. costus* exhibits multiple modes of action, including inhibition of the Epidermal Growth Factor Receptor (EGFR) and PI3K/Akt signaling pathways, as well as suppression of inflammation, invasion, and metastasis. EGFR dysregulation is linked to tumor progression. Thereover, targeting EGFR suppresses cancer cell proliferation 79. Additionally,* S. costus* downregulates TNF-α and NF-κB, key mediators of tumor metastasis and chronic inflammation 80. The ability to modulate multiple pathways makes *S. costus* a promising multi-targeted anticancer agent.

*L. sativum* has been evaluated in fourteen studies on different cancers, highlighting its potential versatility. The anticancer potency varied depending on extraction solvent and methodology. For instance, Nazir et al., (2021) observed differential activity across A-549 and HepG2 cell lines using various organic solvents 64. Furthermore, the selectivity inhibition of the cancer cells has been tested in a study by Ibrahim et al. (2023), who utilized glucosinolates with *L. sativum* ethanol extracts 59. The IC_50_ values for these glucosinolate-treated extracts ranged from 38.5 to 92.6 µg/mL and were noted to have no adverse effects on normal cell lines. While some nanoparticle-based formulations improved bioactivity 57, others, like Ag-MgO nanoparticles, showed no enhancement 55, suggesting that nanoparticle composition critically influences therapeutic efficacy.

Although most *L. sativum* studies were *in vitro*, one *in vivo* study demonstrated a 37.14% increase in lifespan in mice with Ehrlich ascites carcinoma treated with *L. sativum* seed extracts 81. *L. sativum* extracts induced apoptosis and cell cycle arrest—key processes in cancer treatment 59. Apoptosis, a programmed cell death, is regulated at the genetic level, ensuring the orderly and efficient removal of damaged cells 82. Thus, the ability of *L. sativum* extracts to induce the apoptosis underlying its potential as a therapeutic agent.

*R. tripartite* has demonstrated cytotoxic effects. Methanol extracts exhibited the lowest IC_50_ values, particularly against the DLD-1 colon adenocarcinoma cell line (39.83 μg/mL) 68. Aqueous extracts, on the other hand, showed less cytotoxicity, reaffirming the importance of solvent selection. The primary mechanism identified involves inhibition of the PI3K/AKT/mTOR pathway 70. The PI3K/AKT/mTOR pathway plays a crucial role in survival, growth, and proliferation of the cells. Inhibition of this pathway results in suppression of the tumor progression 83.

The cytotoxic potential of *P. communis* was moderate and varied between studies, even though both used fruit extracts. IC₅₀ values ranged from 30.9 to 105 μg/mL in one study 72; While the other reported lower cytotoxicity (IC₅₀: 0.4-3.2 mg/mL), possibly due to differences in extraction methods (hydroinstillation vs. UPLC-PDA-MS) 73. While the findings indicate promising activity, the limited number of studies necessitates further investigation to validate their efficacy against cancer.

In contrast, *C. murale* demonstrated weak anticancer activity, with IC₅₀ values of 1504 μg/mL (MCF-7) and 1267 μg/mL (liver cancer) 74.These values indicate a relatively weak cytotoxic potential, which may limit its applicability in cancer therapy. However, further research using different extraction approaches or targeting other cancer types may yield better results.

*E. hispanica* showed moderate cytotoxic activity in one study, with IC_50_ values ranging from 14.7 μg/mL to 21.4 μg/mL across different cancer cell lines^10^. Similarly, *T. hamosa* demonstrated promising cytotoxicity (IC₅₀ = 6.7-13.7 μg/mL), though only one study has investigated its potential 75. The limited data highlights the necessity for further investigation into these plants to better understand their anticancer potential, considering the influence of plant parts, different solvents and mechanism of action.

This systematic review has several important limitations. First, the literature search was conducted exclusively using Google Scholar, which may not provide the same level of indexing rigor or coverage as more specialized scientific databases. This may have led to the omission of relevant studies. Second, the review only included studies published in English, introducing potential language bias. Another major limitation is the lack of formal risk of bias assessment of the included studies, which undermines the ability to critically evaluate the quality and reliability of the evidence. Furthermore, there was considerable heterogeneity in study designs, plant parts used, extraction methods, solvents, cancer types, and assay conditions, which makes it difficult to compare findings or draw definitive conclusions. Lastly, while some *in vivo* studies were included, the review is heavily weighted toward* in vitro* data, limiting the applicability of findings to clinical or physiological settings.

## 5. Conclusion

Cancer remains a complex, multifactorial disease that is difficult to treat. For decades, using plants as a source of potential therapeutic agents has been a key approach. This systematic review highlights promising anticancer properties of several plant extracts, particularly *S. costus, L. sativum,* and* R. tripartite,* due to their ability to induce apoptosis. Extensive *in vitro* and *in vivo* studies on *S. costus* have demonstrated its significant cytotoxic potential. For *S. costus in vitro* studies, the polarity of the extraction solvents played a significant role in cytotoxic potency. *L. sativum* and *R. tripartita* also demonstrated notable activity, other plants—such as *P. communis, C. murale, E. hispanica,* and* T. hamosa*—require further exploration. Importantly, *A. ochroleuca* and* G. odoratum* have not been studied at all in this context and represent important gaps in literature.

Overall, this review underscores the therapeutic promise of several medicinal plants in cancer treatment and emphasizes the need for standardized methodologies, in vivo studies, and deeper mechanistic investigations to fully harness their potential.

## Figures and Tables

**Table 1:** Comprehensive overview of anticancer studies on selected medicinal plants.

Plant Name	Number of Studies Identified	Types of Experimental Approach
*S. costus*	Forty studies	*In vitro,* and* in vivo*
*L. sativum*	Fifteen studies	*In vitro,* and* in vivo*
*R. tripartite*	Four studies	*In vitro*
*P. communis*	Two studies	*In vitro*
*C. murale*	One study	*In vitro*
*E. hispanica*	One study	*In vitro*
*T. hamosa*	One study	*In vitro*
*A. ochroleuca*	No study	Not applicable
*G. odoratum's*	No study	Not applicable

*No study: No reported studies available for these plants.

**Table 2:** Comprehensive analysis of *in vitro* anticancer studies on* Saussurea costus:* cell lines, extracts, and mechanisms of action.

References	Results Description	Proposed Mechanisms	IC_50_	Extraction Method	Part Used	Cancer Type
Breast Cancer						
34	Decrease the cellular proliferation and control the cancer invasiveness	Inhibition of NF-κB and MMP-9	10 μg/mL	Ethanol by maceration liquid-liquid extraction	Roots	MCF-7
44	Decrease cellular proliferation	Apoptosis regulations	20 μg/mL	Ethanol		
43	Decrease cellular proliferation	Apoptosis by induction of ROS	80 μg/mL	Methanol by soxhlet extraction + MgO nanoparticles		
78	Decrease cellular proliferation	Apoptosis regulations and cell cycle effects	122.5 μg/mL	Methanol by maceration		
28	Decrease cellular proliferation	Not determined	1.7-6.1 µg/mL	Water, Methanol by sonication, Ethyl Acetate by liquid-liquid extraction		
30	Decrease cellular proliferation	Apoptosis regulations and caspase activity	0.54 - 25.5 µg/mL	Methanol, Hexane, Chloroform, Ethyl Acetate, n-Butanol by liquid-liquid extraction	Leaves	
84	Decrease cellular proliferation,	Inhibition of NF-κB and MMP-7	0.5 mg/mL	Aqueous extracts by maceration	Fruits	
42	Decrease cellular proliferation,	Not determined	0.46 μg/mL	Supercritical Carbon Dioxide Extraction	Powder	
39	Decrease cellular proliferation,	Intrinsic apoptosis and mitophagy activation	30.16 μM	Single isolated metabolite (costunolide)	Not applicable	
14	Decrease cellular proliferation,	Not determined	26.7 - 114.6 µg/mL	Palladium nanoparticle with aqueous extracts	Entire plant	
37	Decrease cellular proliferation,	Not determined	5.353 - >100 μg/mL	Ethanol by maceration, Hexane, Chloroform, n-Butanol by liquid-liquid extraction	Roots	MDA-MB-231
34	Decrease the cellular proliferation and control the cancer invasiveness	Inhibition of NF-κB and MMP-9	Ethanol extracts did not inhibit 50% of cells	Ethanol by maceration liquid-liquid extraction		
47	Decrease the cellular proliferation and control the cancer invasiveness	TNFα and NF-κB inhibition	50 μg/mL	Ethanol by sonication		
26	Decrease cellular proliferation	Not determined	0.56 - 0.88 μM/ml	Hexane by soxhlet for 72 hours		
15	Decrease cellular proliferation	Not determined	21.5 µM	Single isolated metabolite (dehydrocostuslactone)	Not applicable	
39	Decrease cellular proliferation	Intrinsic apoptosis and mitophagy activation	12.76 μM	Single isolated metabolite (costunolide )	Not applicable	SK-BR-3
15	Decrease cellular proliferation	Not determined	25.6 µM	Single isolated metabolite (dehydrocostuslactone)	Not applicable	
15	Decrease cellular proliferation	Not determined	43.2 µM	Single isolated metabolite (dehydrocostuslactone)	Not applicable	MDA-MB-453
Colon Cancer						
37	Decrease cellular proliferation	Not determined	4.717->100 μg/mL	Ethanol by maceration, Hexane, Chloroform, n-Butanol (liquid-liquid extraction)	Roots	HCT-116
35	Decrease cellular proliferation	Apoptosis regulation and angiogenesis reduction	82.64 μg/mL	Ethanol and Hexane by cold percolation		
21	The extracts did not affect the cellular viability (reduced cells ~ 20%, however it induced apoptosis	Cell Cycle Arrest, Gene Expression Changes (BCL2., CASP3, TP53, BAX), Mitochondrial Dysfunction	Hexane extracts did not show effect on the viability	Hexane by sonication		
30	Decrease cellular proliferation	Apoptosis regulation and caspases activities modulation	0.4-24.9 µg/mL	Methanol, Hexane, Chloroform, Ethyl acetate, n-Butanol by liquid-liquid extraction	Leaves	
42	Decrease cellular proliferation	Not determined	0.44µg/mL	Supercritical Carbon Dioxide Extraction	Powder	
14	Decrease cellular proliferation, nanoparticles formulation enhances the anticancer activity	Not determined	7.8 - 82.5 µg/mL	Palladium nanoparticles	Not determined	
84	Decrease cellular proliferation	Apoptosis	1 mg/mL	Aqueous extracts by maceration	Fruits	CaCo-2
46	Decrease cellular proliferation	Not applicable	1.16 - 1.55 μM	Methanol	Roots	HCT-15
21	Decrease cellular proliferation	Apoptosis regulation, caspase activity	6.20 - 15.78 µM	Bilosome-based on single isolated metabolites (costunolide)	Not applicable	LS174T
21	The extracts did not affect the cellular viability (reduced cells ~ 20%, however it induced apoptosis	Cell Cycle Arrest, Gene Expression Changes (BCL2., CASP3, TP53, BAX), Mitochondrial Dysfunction,	The hexane extracts did not show effect on the viability	Hexane by sonication	Roots	HT-29
Liver Cancer						
36	Decrease cellular proliferation	Apoptosis regulation, caspase activity	56.76 µg/mL	Chloroform, n-Butanol, Ethyl Acetate by soxhlet apparatus	Roots	HepG2
35	Decrease cellular proliferation	Apoptosis and reduction of the angiogenesis	154.30 μg/mL	Ethanol, Hexane by cold percolation		
85	Decrease cellular proliferation	Apoptosis regulation	1.10 - 3.5 mg/mL	Aqueous, Ethanol, Hydro-ethanol		
86	Decrease cellular proliferation	Apoptosis regulation and reactive oxygen species induction	20 mM	Methanol, Water, Petroleum Ether, n-Butanol, Acetone, Ethyl Acetate		
36	Decrease cellular proliferation	Apoptosis regulation, caspase activity	56.76 µg/mL	Chloroform, n-Butanol, Ethyl Acetate by soxhlet apparatus		
87	Decrease cellular proliferation	Autophagy inhibition	5 - 20 μg/mL	Hexane, Ethyl Acetate, n-Butanol, Water		
86	Decrease cellular proliferation	Apoptosis regulation and reactive oxygen species induction	20 mM	Methanol, Water, Petroleum Ether, n-Butanol, Acetone, Ethyl Acetate		
30	Decrease cellular proliferation	Apoptosis regulation, caspase activity	0.5-33.2 µg/mL	Methanol, Hexane, Chloroform, Ethyl Acetate, n-Butanol by liquid-liquid extraction	Leaves	
42	Decrease cellular proliferation	Not determined	0.74 μg/mL	Supercritical Carbon Dioxide	Powder	
18	Decrease cellular proliferation	Intrinsic apoptosis, ER stress induction, MAPK activation, Phosphorylation signaling	16.7 µM	Single isolated metabolite (dehydrocostuslactone)	Not applicable	
14	Decrease cellular proliferation, nanoparticles formulation enhances the anticancer activity	Not determined	11.8 - 91.5 µg/mL	Palladium nanoparticles	Not applicable	
18	Decrease cellular proliferation	Intrinsic apoptosis, ER stress induction, MAPK activation, Phosphorylation signaling; Apoptosis	18.8 µM	Single isolated metabolite (dehydrocostuslactone)	Not applicable	PLC/PRF/5
86	Decrease cellular proliferation	Apoptosis regulation and reactive oxygen species induction	20 µM	Methanol, Water, Petroleum Ether, n-Butanol, Acetone, Ethyl Acetate	Roots	SMMC-7721
Lung Cancer						
37	Decrease cellular proliferation	Not determined	11.875->100 μg/mL	Ethanol by maceration, Hexane, Chloroform, n-Butanol by liquid-liquid extraction	Roots	A-549
23	Decrease cellular proliferation	Not determined	38.5->100 µg/m	Ethanol, Chloroform, Ethyl Acetate, n-Butanol		
46	Decrease cellular proliferation	Not determined	1.64-2.97 μM	Methanol		
17	Decrease cellular proliferation	Not determined	37.90 µg/ml	Chloroform, Ethanol by soxhlet apparatus		
26	Decrease cellular proliferation	Not determined	3.9 - 7.4 μM/ml	Hexane by soxhlet apparatus for 72 hours		
40	Decrease cellular proliferation	Gene expression activity (BCL2, P53, BAX), suppression of the TNFα and NF-κB	6.1 -13.4 µM	Single isolated metabolites (costunolide)	Not applicable	
20	Decrease cellular proliferation	Cell cycle affects, Apoptosis regulation, Protein expression changes (p53, p27, p21)	~50-60 µM	Single isolated metabolites (costunolide)	Not applicable	SK-MES-1
Neuroblastoma						
26	Decrease cellular proliferation	Not determined	4.1 - 4.2 μM/mL;	Hexane by soxhlet apparatus for 72 hours	Roots	IMR-32
32	Decrease cellular proliferation and control the cancer invasiveness	Apoptosis regulation and reduction the invasion of the cell	1.26-6.52 μM	Two isolated metabolites (dehydrocostus lactone and costunolide)	Not applicable	
46	Decrease cellular proliferation	Not determined	0.43 - 1.70 μM;	Methanol;	Roots;	XF498
29	Decrease cellular proliferation	Apoptosis regulation, caspase activity, gene expression activity (BCL2, P53, BAX), changes in protein expression (AKT and GSK-3β activity)	15 μg/mL	Ethanol	Roots	SH-SY5Y
29	Decrease cellular proliferation	Apoptosis regulation, caspase activity, gene expression activity (BCL2, P53, BAX), changes in protein expression (AKT and GSK-3β activity)	20 μg/mL	Ethanol	Roots	B103
32	Decrease cellular proliferation and control the cancer invasiveness	Apoptosis regulation and reduction the invasion of the cell	1.26-6.52 μM	Two isolated metabolites (dehydrocostus lactone and costunolide)	Not applicable	LA-N-1
32	Decrease cellular proliferation and control the cancer invasiveness	Apoptosis regulation and reduction the invasion of the cell	1.26-6.52 μM	Two isolated metabolites (dehydrocostus lactone and costunolide)	Not applicable	SK-N-SH
Prostate Cancer						
26	Decrease cellular proliferation	Not determined	0.64 - 3.4 μM/ml	Hexane by soxhlet apparatus for 72 hours	Roots	DU-145
21	Decrease cellular proliferation	Apoptosis regulation, caspase activity, gene expression activity (BCL2, P53, BAX)	Hexane extracts did not affect the viability	Hexane by sonication		
38	The extracts did not affect the cellular viability (reduced cells ~ 20%, however it induced apoptosis and inhibits the cell migration	Changes in TIMP, MMP-9 expression, inhibition of cell migration	Hexane did not affect the viability of the cell			
33	Decrease cellular proliferation	Apoptosis regulation, gene expression activity (BCL2, P53, BAX), effects on androgen signaling, decrease cell migration, effects on the autophagy activity	50 µg/ml	Ethanol	Roots	LNCaP
21	Decrease cellular proliferation	Apoptosis regulation, caspase activity, gene expression activity (BCL2, P53, BAX)	Hexane extracts did not affect the viability	Hexane by sonication		
38	Decrease cellular proliferation	Not determined	3.37 - >100 μg/mL	Ethanol by maceration, Hexane, Chloroform, n-Butanol (liquid-liquid extraction)	Roots	PC-3
38	The extracts did not affect the cellular viability (reduced cells ~ 20%, however it induced apoptosis and inhibits the cell migration	Changes in TIMP, MMP-9 expression, inhibition of cell migration	Hexane did not affect the viability of the cell	Hexane by sonication	Roots	TRAMP-C
Gastric Cancer						
45	Decrease cellular proliferation	Apoptosis regulation, caspase activity, gene expression activity (BCL2, P53, BAX), cell cycle effects	100 μg/mL	Ethanolic extracts by sonication followed by freeze-drying	Roots	AGS
31	Decrease cellular proliferation	Apoptosis regulation, caspase activity, gene expression activity (BCL2, P53, BAX), cell cycle effects	79 μg/mL	Ethanolic extracts by sonication followed by freeze-drying		
41	Decrease cellular proliferation	Not determined	4.5 μM	Single isolated metabolites (costunolide)	Not applicable	
Leukemia						
22	Decrease cellular proliferation	Apoptosis regulation, cell cycle effects, and decrease the proteins expression of the multidrug resistance	Not determined	Methanol; Petroleum Ether, Methanol by soxhlet apparatus	Roots	CCRF-CEM
25	Decrease cellular proliferation	Apoptosis and NF-κB inhibition	5 mM	Partitioning into Ethyl Acetate, Water, n-Butanol	Roots	HL-60
46	Decrease cellular proliferation	Apoptosis and Cell cycle effects	Not determined	Methanol	Roots	U937
Cervical Cancer						
26	Decrease cellular proliferation	Not determined	1.1 - 2.4 μM/mL	Hexane by soxhlet apparatus for 72 hours	Roots	SIHA
87	Decrease cellular proliferation	Apoptosis regulation and reactive oxygen species induction	20 µM	Methanol, Water, Petroleum Ether, n-Butanol, Acetone, Ethyl Acetate	Roots	HeLa
Esophageal Cancer						
27	Decrease cellular proliferation and inhibit cell migration	Migration Inhibition, Prefoliation Prevention, Apoptosis Induction; Inhibition of the cell migration and expression of MMp-2 and MPP-9, modulation of the autophagy process, Induction of ROS	10.55 - 43.75 µM	Ethanol by maceration, Petroleum Ether by liquid-liquid extraction	Roots	Eca109
27	Decrease cellular proliferation and inhibit cell migration	Migration Inhibition, Prefoliation Prevention, Apoptosis Induction; Inhibition of the cell migration and expression of MMp-2 and MPP-9, modulation of the autophagy process, Induction of ROS	6.97 - 24.29 μg/mL	Hydro-distillation using a Clevenger-type device		
27	Decrease cellular proliferation and inhibit cell migration	Migration Inhibition, Prefoliation Prevention, Apoptosis Induction; Inhibition of the cell migration and expression of MMp-2 and MPP-9, modulation of the autophagy process, Induction of ROS	8.35 - 40.78 µM	Ethanol by maceration, Petroleum Ether by liquid-liquid extraction	Roots	KYSE150
Ovarian Cancer						
46	Decrease cellular proliferation	Not determined	1.65 - 1.83 μM	Methanol	Roots	SK-OV-3
15	Decrease cellular proliferation	Not determined	10.8 μM	Single isolated metabolite (dehydrocostus lactone)	Not applicable	
15	Decrease cellular proliferation	Not determined	13.9 μM	Single isolated metabolite (dehydrocostus lactone)	Not applicable	OVCAR3
Colorectal Cancer						
16	Decrease cellular proliferation	Apoptosis regulation, caspase activity, suppression of the TNFα and NF-κB, suppression of the Suppression of Nuclear Translocation	5nM	Two isolated metabolites (dehydrocostus lactone and costunolide)	Not applicable	SW-480
Oral Cancer						
24	Decrease cellular proliferation	Apoptosis regulation, caspase activity	30 µg/mL	Methanol	Roots	KB
Skin Cancer						
46	Decrease cellular proliferation	Not determined	0.55- 0.59 μM	Methanol	Roots	SK-MEL-2
Soft Tissue Sarcoma						
22	Decrease cellular proliferation	Apoptosis regulation, caspase activity, gene expression activity (BCL2, P53, BAX)	7.41 - 9.71 μg/mL	Methanol; Petroleum Ether, Methanol by soxhlet apparatus	Roots	SW-872
22	Decrease cellular proliferation	Apoptosis regulation, caspase activity, gene expression activity (BCL2, P53, BAX)	6.17 - 9.61 μg/mL	Methanol; Petroleum Ether, Methanol by soxhlet apparatus	Roots	SW-982
22	Decrease cellular proliferation	Apoptosis regulation, caspase activity, gene expression activity (BCL2, P53, BAX)	8.33 - 9.75 μg/mL	Methanol; Petroleum Ether, Methanol by soxhlet apparatus	Roots	TE-671
Pancreatic Cancer						
26	Decrease cellular proliferation	Not Determined	0.26 - 1.2 μM/mL	Hexane	Roots	PANC1

*Not applicable: The studies did not extract the compound, they tested the isolated compounds, which are mainly (dehydrocostus lactone or costunolide). *Not determined: The studies did not investigate the mechanism of action of anticancer activity.

**Table 3:** Comprehensive analysis of *S. costus* reported *in vivo* studies: treatment protocols and observed effects.

Cancer Type	Animal Model	Part Used	Extraction Method	Dose	Proposed Mechanisms	Results Description	References
Breast Cancer							
MCF-7	Adult Sprague Dawley (SD) rats (sex-female; weight-160 ±20 g; age-6-8 weeks	Roots	Ethanol by sonication	(100, 250 and 500 mg/kg BW)	Not determined	Inhibits the pulmonary metastases breast cancer	51
	Six-week-old nude (Nu/Nu) mice	Not applicable	Single isolated metabolite (costunolide)	20 μM Three times a week for 30 days	Suppress breast cancer growth and metastases by inhibiting TNFα-induced NF-κB activation	Inhibits tumor growth and prevent migration	47
MDA-MB-23	Female BALB/c nude mice (4 weeks old)	Roots	Hexane by sonication	20 mg/kg/day	Cell cycle arrest and apoptosis regulations	Inhibits tumor growth	88
Liver Cancer							
Not determined	Albino Swiss mice (aged 8-10 weeks, with an average body weight of 28±1.5 g)	Roots	Ethanol	400, 600, 800mg/Kg	Cell cycle arrest and apoptosis regulations	Inhibits tumor growth	48
SMMC-7721	Male nude mice (4 weeks old; BALB/c-nude)	Roots	Hexane	15 mg/kg/day	Apoptosis and anti-metastatic activity.	Inhibits tumor growth	52
Lung Cancer							
LC-540	Adult Sprague Dawley (SD) rats (sex-female; weight-160 ±20 g; age-6-8 weeks)	Roots	Ethanol	100, 250 and 500 mg/kg BW	Not determined	Inhibits tumor growth	89
Leukemia							
Not determined	Adult male albino rats (180-220g); Male nude mice (4 weeks old; BALB/c-nude)	Roots	Ethanol	(300mg/Kg/day) orally for 4 weeks	Not determined	Inhibits tumor growth	50
Laryngeal Cancer							
Not determined	Female nude mice (BALB/c nu/nu, 4-5 weeks old, 18-19 g)	Roots	Ethanol by maceration	10, 15 mg/kg	Inhibition of PI3K/Akt/Bad pathway	Inhibits tumor growth	90
Esophageal Cancer							
Not determined	SPF-grade female BALB/c nude mice aged 4-5 weeks	Roots	Ethanol by maceration, Petroleum Ether by liquid-liquid extraction	(0, 20, and 40 mg/kg/day) for 28 days	Migration Inhibition, Prefoliation Prevention, Apoptosis Induction; Inhibition of the cell migration and expression of MMp-2 and MPP-9, modulation of the autophagy process, Induction of ROS	Inhibits tumor growth and migration	27
Gastric Cancer							
MKN-28	Female BALB/c nude mice each weighing 20 g ± 2 g	Not applicable	Single isolated metabolite (dehydrocostus lactone)	15, 30 mg/kg/day	Inhibition of autophagy	Inhibits tumor growth	49

*Not applicable: The studies did not extract the compound, they tested the isolated compounds, which are mainly (dehydrocostus lactone or costunolide. *Not determined: The studies did not determine the type of cell line, or did not investigate the mechanism of action of anticancer activity.

**Table 4:** Comprehensive analysis of *in vitro* anticancer studies on *L. sativum:* cell lines, extracts, and mechanisms of action.

Cancer Type	Plant Part	Extraction Method	IC_50_	Proposed Mechanism	Results Description	Reference
Liver Cancer						
HepG-2	Seeds	Methylene chloride, n-Hexane, Ethyl Acetate, Butanol, Methanol	45 - 63.8 µg/mL	Apoptosis and downregulation of EGFR	Decrease cellular proliferation	64
		Aqueous with ZnO nanoparticles	100 µg/mL	Induction of ROS	Decrease cellular proliferation	63
	Seeds and leaf	Ethanol and Aqueous	382.2 mg /mL	Not determined	Decrease cellular proliferation	58
	Leaves and roots	Ethanol extracts with glucosinolate	38.5 - 81.2 µg/mL	Apoptotic regulation, caspase activity, cell cycle effects	Decrease cellular proliferation, the glucosinolate extracts enhance the selectivity, it did not affect normal cell lines	59
HuH-7	Seeds	Methylene chloride, n-Hexane, Ethyl Acetate, Butanol, Methanol;	59 - 63.5 µg/mL	Gene expression modulation (EGFR, BCL2, SMAD3, BAX, P53)	Decrease cellular proliferation	64
Breast Cancer						
MCF-7	Seeds	Aqueous	70% concentration of *L. sativum* inhibited the growth by 64.03%	Not determined	Decrease cellular proliferation	62
		Crude and soxhlet Methanol	88.49 - 136.75 µg/mL	Not determined	Decrease cellular proliferation	65
	Leaves and Roots	Ethanol extracts with glucosinolates	61 - 71.1 µg/mL	Apoptosis regulation, caspase activity, cell cycle effects	Decrease cellular proliferation, the glucosinolate extracts enhance the selectivity, it did not affect normal cell lines	59
Colon Cancer						
CaCo-2	Seeds	Aqueous extract with ZnO-NPs	18.45 - 105.9 µg/mL	Gene expression modulation (p53, Bax, Bcl-2	Decrease cellular proliferation	57
	Leaves	Ethanol extracts with glucosinolates	56.6 - 89.9 µg/mL	Apoptotic regulation, caspase activity, cell cycle effects	Decrease cellular proliferation, the glucosinolate extracts enhance the selectivity, it did not affect normal cell lines	59
DLD-1	Above-Ground Parts	Methanol by maceration	100 µg/mL (DLD-1)	100 µg/mL	Decrease cellular proliferation	66
HT-29	Seeds	n-Hexane, Chloroform, Ethyl Acetate Methanol by soxhlet apparatus	100 µg/mL	Not determined	Decrease cellular proliferation	60
HT-15	Seeds	n-Hexane, Chloroform, Ethyl Acetate Methanol by soxhlet apparatus	100 µg/mL	Not determined	Decrease cellular proliferation	60
Cervical Cancer						
HeLA - 2	Seeds	Soxhlet extraction with silver nanoparticles	135 - 220.35 μg/mL	ROS generation	Decrease cellular proliferation, and nanoparticles formulation enhances the anticancer activity	55
	Leaves	Ethanolic maceration extracts	100 µg/mL (at second day)	Apoptosis induction	Decrease cellular proliferation	61
Lung Cancer						
A-549	Seeds	n-Hexane, Chloroform, Ethyl Acetate Methanol by soxhlet apparatus	100 µg/mL	Not determined	Decrease cellular proliferation	60
	Leaves and Roots	Ethanol extracts with glucosinolates	42.3 - 92.6 µg/mL	Apoptotic regulation, caspase activity, cell cycle effects	Decrease cellular proliferation, the glucosinolate extracts enhance the selectivity, it did not affect normal cell lines	59
Prostate Cancer						
PC-3	Seeds and leaf Calli	Aqueous and Ethanol extracts	113.6 mg/mL	Not determined	Decrease cellular proliferation	58
	Leaves and Roots	Ethanol extracts with glucosinolates	51.4 - 72.4 µg/mL	Apoptotic regulation, caspase activity, cell cycle effects	Decrease cellular proliferation, the glucosinolate extracts enhance the selectivity, it did not affect normal cell lines	59
Endometrium Cancer						
ECC-1	Above-Ground Parts	Methanol extracts by maceration	353 µg/mL	Not determined	Decrease cellular proliferation	66
Tongue Squamous Carcinoma						
CAL-27	Leaves	Aqueous extract	100 µg/mL	ROS generation induction	Decrease cellular proliferation	54
Melanoma						
A-375	Leaves and roots	Ethanol extracts with glucosinolates	Not determined	Apoptotic regulation, caspase activity, cell cycle effects	Decrease cellular proliferation, the glucosinolate extracts enhance the selectivity, it did not affect normal cell lines	59
Neuroblastoma						
IMR-32	Seeds	Various solvents by soxhlet apparatus	100 µg/mL	Not determined	Decrease cellular proliferation	60
Ovarian Adenocarcinoma						
OV17R	Seeds	Various extracts and HPLC	28.8 - 64.32 μg/mL	Not determined	Decrease cellular proliferation	53
Leukemia						
Jurkat E6-1	Seeds	Tertiary Alkaloid extract	75.25 mg/mL	Apoptosis via DNA laddering, caspase-3 activity	Decrease cellular proliferation	56
Colorectal Cancer						
SW-480	Seeds	Aqueous extract\ green synthesis of ZnO-NP	13.14 - 100 μg/mL	Gene expression modulation (EGFR, BCL2, SMAD3, BAX, P53)	Decrease cellular proliferation	57

*Not determined: The studies did not investigate the mechanism of action of anticancer activity.

**Table 5:** Comprehensive analysis of *L. sativum* reported in* in vivo* studies: treatment protocols and observed effects.

Cancer Type	Animal Model	Part Used	Extraction Method	Dose	Proposed Mechanisms	Results Description	References
Ehrlich ascites carcinoma (EAC)							
Ehrlich ascites carcinoma (EAC)	Female Swiss albino mice	Seeds	Dichloromethane and Ethyl Acetate.	500 mg/kg	Decreased chromosomal aberration and DNA fragmentation induced by EAC in mice	The mice have an increased lifespan by 37.14%.	82

**Table 6:** Comprehensive analysis of *in vitro* anticancer studies on *R. tripartite:* cell lines, extracts, and mechanisms of action.

Cancer Type	Part Used	Extraction Method	IC_50_	Proposed Mechanisms	Results Description	References
Acute Myeloid Leukemia						
THP-1	Leaves	Acetone, Methanol buy maceration	63 μg/mL	Apoptosis induction by inhibiting PI3K/AKT/mTOR signaling pathway.	Decrease cellular proliferation	70
K-562	Aerial Parts	Acetone, Methanol buy maceration	42.89 μg/mL	Not determined	Decrease cellular proliferation	71
Colon Adenocarcinoma						
DLD-1	Roots	Hexane, Dichloromethane, Methanol, Water by soxhlet extraction	39.83 - 200 μg/mL	Not determined	Decrease cellular proliferation	68
CaCo-2	Aerial parts	Acetone, Methanol buy maceration	44.87 μg/mL	Not determined	Decrease cellular proliferation	71
Breast Adenocarcinoma						
MCF-7	Roots	Ethanol by maceration, then sequential partitioning: Hexane, Ethyl acetate, n-butanol	100 μg/mL	Not determined	Decrease cellular proliferation	69
Lung Cancer						
A-549	Roots	Hexane, Dichloromethane, Methanol, Water by soxhlet extraction	60.69 - 205.52 μg/mL	Not determined	Decrease cellular proliferation	68

*Not determined: The studies did not investigate the mechanism of action of anticancer activity.

**Table 7:** Comprehensive analysis of *in vitro* anticancer studies on *P. communis:* cell lines, extracts, and mechanisms of action.

Cancer Type	Part Used	Extraction Method	IC_50_	Proposed Mechanism	Results Description	References
Lung Cancer						
A-549	Fruits	Hydroinstillation	30.9 μg/mL	Not determined	Decrease cellular proliferation	72
	Fruits	UPLC-PDA-MS	0.5 - 2.5 mg/mL	Not determined	Decrease cellular proliferation	73
WI-38	Fruits	Hydroinstillation	55.9 μg/ml	Not determined	Decrease cellular proliferation	72
Colon Cancer						
HT-29	Fruits	UPLC-PDA-MS	0.5 - 2.5 mg/mL	Not determined	Decrease cellular proliferation	73
Breast Cancer						
MCF-7	Fruits	UPLC-PDA-MS	0.4 - 2.4 mg/mL	Not determined	Decrease cellular proliferation	73
Prostate Cancer						
LNCaP	Fruits	UPLC-PDA-MS	0.5 - 1.4 mg/mL	Not determined	Decrease cellular proliferation	73
Urinary Cancer						
HCV29T	Fruits	UPLC-PDA-MS	0.5 - 1.5 mg/mL	Not determined	Decrease cellular proliferation	73
Kidney Cancer						
A498	Fruits	UPLC-PDA-MS	1.8 - 3.2 mg/mL	Not determined	Decrease cellular proliferation	73
Ovary Cancer						
CHO-K1	Fruits	Hydroinstillation	105 μg/mL	Not determined	Decrease cellular proliferation	72
Leukemia						
M-NFS-60	Fruits	Hydroinstillation	56.5 μg/mL	Not determined	Decrease cellular proliferation	72

*Not determined: The study did not investigate the mechanism of action of anticancer activity.

**Table 8:** Comprehensive analysis of *in vitro* anticancer studies on *C. murale:* cell lines, extracts, and mechanisms of action.

Cancer Type	Part used	Extraction Method	IC_50_	Proposed Mechanisms	Results Description	References
Breast Cancer						
MCF-7	Leaves	Ethanol extracts microwave assisted extraction.	1504 µg/mL	Not determined	Decrease cellular proliferation	74
Liver Cancer						
HCAM	Leaves	Ethanol extracts microwave assisted extraction.	1267 µg/mL	Not determined	Decrease cellular proliferation	74

*Not determined: The studies did not investigate the mechanism of action anticancer activity.

**Table 9:** Comprehensive analysis of *in vitro* anticancer studies on *E. hispanica:* cell lines, extracts, and mechanisms of action.

Cancer Type	Part Used	Extraction and Method	IC_50_	Proposed Mechanism	Results Description	References
Breast Cancer						
MCF-7	Ground, Aerial parts	Methanol	18 μg/mL	Not determined	Decrease cellular proliferation	10
Liver Cancer						
HePG-2	Ground, Aerial parts	Methanol	20.8 μg/mL	Not determined	Decrease cellular proliferation	10
Cervical Cancer						
HeLA-2	Ground, Aerial parts	Methanol	14.7 μg/mL	Not determined	Decrease cellular proliferation	10
Colon Cancer						
HCT-116	Ground, Aerial parts	Methanol	21.4 μg/mL	Not determined	Decrease cellular proliferation	10

* Not determined: The studies did not investigate the mechanism of action of anticancer activity.

**Table 10:** Comprehensive analysis of *in vitro* anticancer studies on *T. hamosa:* cell lines, extracts, and mechanisms of action.

Cancer Type	Part Used	Extraction and Method	IC_50_	Proposed Mechanism	Results Description	References
Breast Cancer						
MDA-MB-231	Aerial Parts	Methanol	28.9 μM	Not determined	Decrease cellular proliferation	75
Lung Cancer						
A-549	Aerial Parts	Methanol	21.2 μM	Not determined	Decrease cellular proliferation	75
Colon Cancer						
HCT-116	Aerial Parts	Methanol	59.1 μM	Not determined	Decrease cellular proliferation	75

*Not determined: The studies did not investigate the mechanism of action of anticancer activity.

## Abbreviations

ALP: alkaline phosphatase
ALT: alanine aminotransferase
AST: aspartate aminotransferase
BUN: blood urea nitrogen
DHE: dehydrocostuslactone
EGF: epidermal growth factor
ER: endoplasmic reticulum
GL: glucosinolates of leaf cell suspension
GLM: glucosinolates of leaf cell suspension treated with L-methionine
GLT: glucosinolates of leaf cell suspension treated with L-tyrosine
GSH: glutathione
LAMP: lysosome-associated membrane protein
LC3II: microtubule-associated protein 1 light chain 3-II lipidation chain indicator
MDA: malondialdehyde
MEK: mitogen-activated protein kinase kinase MAPKK
MMP: matrix metalloproteinase
MS: mass spectrometry
NF-κB: nuclear factor-kappa B
OL: petroleum ether extract of leaf cell suspension
OR: petroleum ether extract of root cell suspension
PARP: poly ADP-ribose polymerase
PDA: photodiode array detector
PI3K/AKT/mTOR: phosphatidylinositol 3-kinase PI3K protein kinase B AKT mammalian target of rapamycin mTOR
RNS: reactive nitrogen species
ROS: reactive oxygen species
TIMP: tissue inhibitor of metalloproteinases
TNF-α: tumor necrosis factor-alpha
UPLC: ultra-performance liquid chromatography
VEGF: vascular endothelial growth factor
